# Urbanization and global warming impacts of Indonesia’s future capital of Nusantara on air temperature and urban heat island

**DOI:** 10.1038/s41598-025-25500-8

**Published:** 2025-11-24

**Authors:** Muhammad Rais Abdillah, Rahastuti Tiara Adysti, Winnilaswita Wijaya, I Dewa Gede Agung Junnaedhi, Nurjanna Joko Trilaksono, Rusmawan Suwarman, Marzuki Marzuki, Rahmat Hidayat, Yahdi I. Miftahuddin, Prawira Yudha Kombara, Huda A. Mukhsinin

**Affiliations:** 1 Atmospheric Science Research Group, Faculty of Earth Science and Technology, Institut Teknologi Bandung, Bandung, Indonesia; 2 Center for Climate Change, Institut Teknologi Bandung, Bandung, Indonesia; 3 Meteorology Study Program, Faculty of Earth Science and Technology, Institut Teknologi Bandung, Bandung, Indonesia; 4 Department of Physics, Universitas Andalas, Padang, Indonesia; 5 Department of Geophysics and Meteorology, IPB University, Bogor, Indonesia; 6 Deputy for Planning and Land Affairs, Nusantara National Capital Authority (OIKN), Balikpapan, Indonesia; 7 National Research and Innovation Agency (BRIN), Research Center for Climate and Atmosphere, Bandung, Indonesia; 8 Indonesia Meteorological Climatological and Geophysics Agency (BMKG), Meteorological Station of Sultan Aji Muhammad Sulaiman Sepinggan, Balikpapan, Indonesia

**Keywords:** Indonesia’s capital of Nusantara, Land use change, Regional climate projection, Global warming, Urban heat island, Surface air temperature, Climate change, Environmental impact

## Abstract

Indonesia is developing a new capital, Ibu Kota Nusantara (IKN), which is expected to become a megacity in Southeast Asia. Although promoted as a sustainable city integrated with tropical ecosystems, its potential impact on future temperature increases remains unclear. This study uses mesoscale climate projections with the official land-use plan and the SSP5-8.5 scenario to assess surface air temperature changes in and around IKN. The model simulated urban heat islands (UHIs) in the western and eastern parts of IKN, with the western UHI being strongest and slightly extending northward to rural areas owing to the southerly background wind. Results show that the global warming exerts a larger effect on temperatures than urbanization. Early nighttime temperatures increase by up to 0.78 °C from urbanization alone, 1.9 °C from global warming, and 2.54 °C when both effects are simulated in the mid-twenty-first century. Low wind conditions further amplifies the warming, especially in upwind regions. The finding provides critical insights for mitigating potential heat stress and refining spatial planning in IKN and its surrounding regions.

**Supplementary Information:**

The online version contains supplementary material available at 10.1038/s41598-025-25500-8.

## Introduction

The relocation of Indonesia’s capital from Jakarta to Nusantara, which is located in the island of Kalimantan, was mandated in 2019 and its detailed plan was published in 2022^1,2^. The new capital, Nusantara (commonly referred to as Ibu Kota Nusantara or IKN), has progressed rapidly since then, although it remains far from complete^3^. The city is ambitiously designed with a large green fraction and to achieve net-zero emissions by the mid-twenty-first century. With the support of advanced technology, the city is aiming to be recognized as a smart sustainable city in future^4^.

As the new capital is not built from an existing city but from forest area, the city construction will inevitably transform the land cover. The built-up or urban cover area will gradually increase due to urbanization. The presence of buildings in the planned city may alter the climate characteristics owing to changes in heat fluxes, radiation exchange, surface thermal, roughness, and anthropogenic heat emission. These changes can lead to increased air temperature, reduced wind speed, and modified moisture and precipitation^5–7^. The urban temperature, which is warmer than its surrounding rural areas, yields a phenomenon called urban heat island (UHI)^8^, whose features vary spatially and temporally depending on the local day-night cycle and seasonal conditions^6^. Furthermore, UHI may extend as an urban “plume” due to wind advection^9^. The extension spreads the urban air —which is typically hotter, more humid, and more polluted— to areas outside the city and thus potentially increases environmental risks in suburbs or rural areas. Oke et al.^6^ provides a comprehensive textbook on urban climate and UHI.

Capital relocations have been shown to significantly modify land use/land cover (LULC), consequently affecting local climate and ecosystems. When Brazil moved its capital from Rio de Janeiro to Brasília in 1960, the city was built in a wooded savanna with limited forest cover^10^, yet the resulting urbanization still produced distinct warming and a prominent nighttime urban heat island (UHI)^11,12^. In Nigeria, satellite observations indicate that Abuja has experienced sharp declines in vegetation cover since its designation as the capital in 1991^13^, driving consistent warming over the city^14^. In Southeast Asia, the construction of Naypyidaw in Myanmar removed hundreds of km^2^ of tropical forest, leading to notable surface warming^15,16^. Assessing such impacts before a city is built, particularly under future climate change, is essential for developing effective and sustainable mitigation strategies.

When comparing the effects of urbanization and global warming on rising temperatures, previous studies showed that local LULC changes can contribute to greater warming than the global effect, exabberating as shown, for example, in some parts of the Greater Jakarta^17^. Such pronounced effects of LULC change highlight shortcomings in sustainable urban design, leading to amplified warming and intensified UHI. The new capital is designed with a large proportion of green space and dispersed patches of vegetation to mitigate these risks^18^. However, the effectiveness of this strategy in suppressing future warming remains uncertain due to the lack of related studies. Most climate-related publications on the new capital have focused on hydrometeorological issues rather than temperature increases^19–27^. Furthermore, these studies mostly relied on historical observations without accounting for global warming or potential LULC changes in future.

The study examines the future impacts of urbanization and global warming on Indonesia’s planned new capital. By leveraging a regional climate model and incorporating the updated official LULC data, we focus our attention on the responses of air temperature, UHI, and their variations under the influence of wind. The study is intended to test the effectiveness of IKN’s sustainable city design by evaluating whether the impact of LULC change is weaker than—or at least comparable to—that of global warming. The findings are expected to support spatial planners in assessing the current grand design, particularly in mitigating heat stress risks. Sect. Data and method provides a detailed explanation of the data and method used in this study. Sect. Reults and discussion presents the results, highlighting the spatial and diurnal variations in atmospheric changes. Sect. Discussion summarizes the findings and discusses the possible future research directions.

## Data and method

Many previous studies of UHI, especially in Indonesia, used intermittent satellite-derived surface temperature data, which actually represent surface urban heat island (SUHI) (e.g.,^28–31^). Although some variations in atmospheric UHI are reflected in SUHI, it is not possible to investigate the direct effects of air temperature and the possible urban plume from SUHI. Since near-surface weather observations are usually limited in terms of availability and spatial representativeness^17,32^, numerical simulations are more preferable to conducting such studies. In addition, the simulations allow us to estimate future climate projections and various LULC scenarios to assess climate change adaptation and mitigation strategies.

This study employs a mesoscale climate model to simulate the combined effects of urbanization and global warming. Two official LULC datasets—representing conditions without and with the new capital design—are used as model inputs to characterize surface conditions before and after urbanization. To incorporate the influence of global warming, we apply a pseudo-global-warming (PGW) approach, in which the differences between future and historical simulations of global models are added to the initial and boundary conditions of the regional model^33,34^. The details of the data and methods are provided below.

### Data

**Fig. 1:**
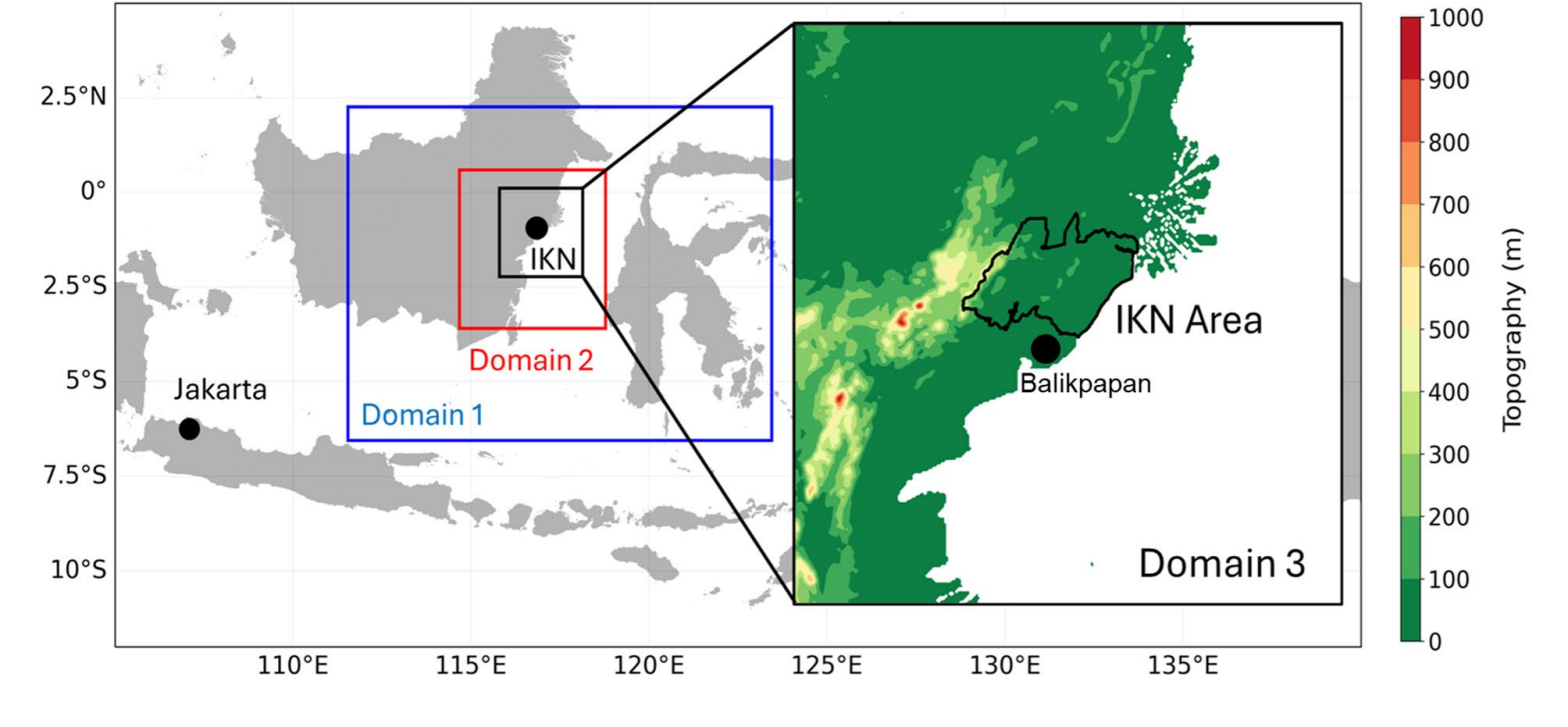
Location of study area. Blue, red, and black boxes denote domain 1 (9 km resolution), domain 2 (3 km), and domain 3 (1 km) of the regional climate model. The panel on the right side shows a topography feature of domain 3 with the new capital of Nusantara or IKN.

**Fig. 2:**
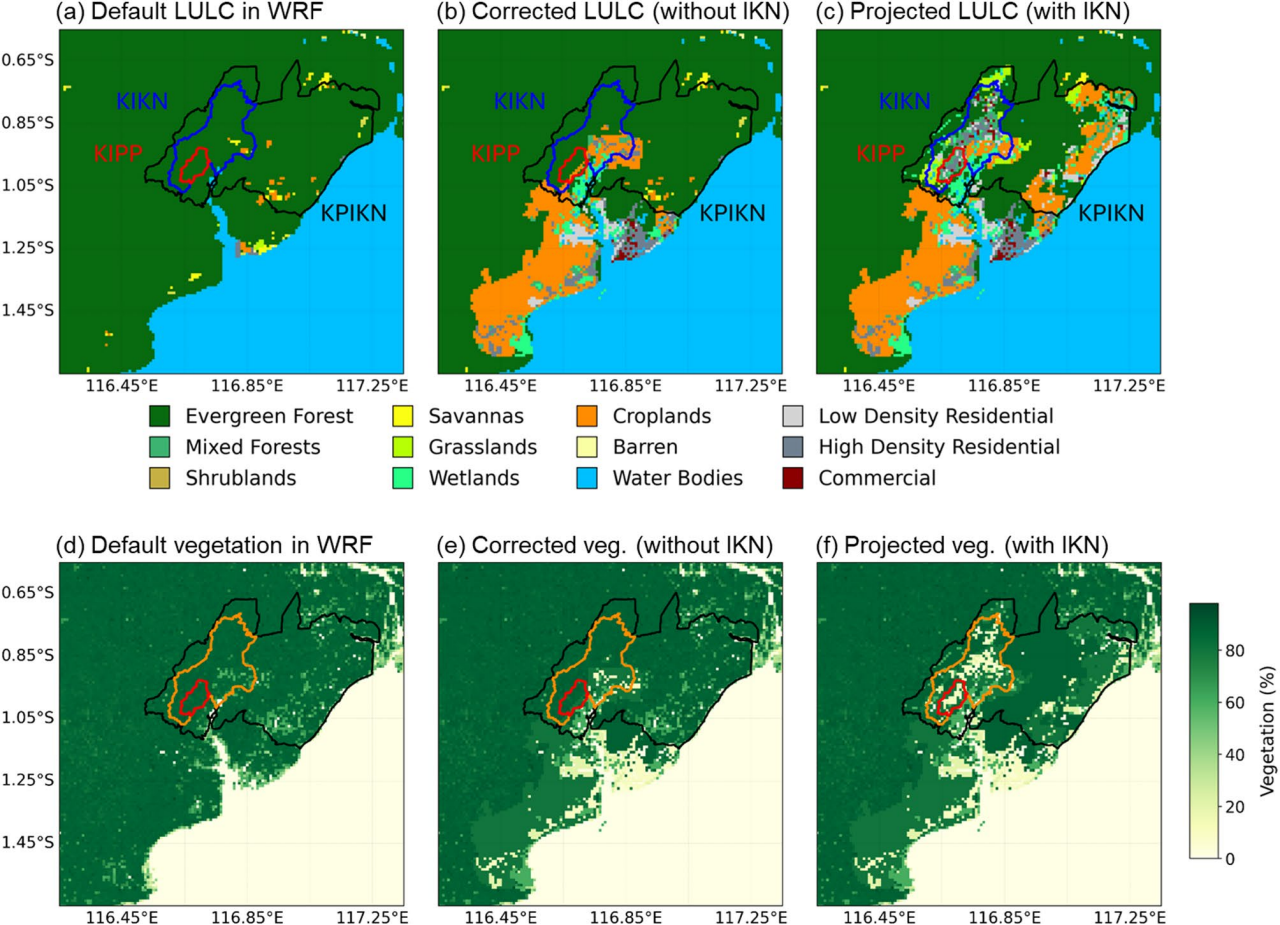
(**a-c**) Land use/land cover (LULC) and (**d–f**) vegetation fraction data used in this study. (**a**, **d**) Original MODIS LULC and vegetation fraction obtained from WRF model. (**b**, **e**) Corrected land surfaces data representing present condition for climate simulations without the new capital. (**c**, **f**) Projected land surfaces data for climate simulations with the new capital. In (**a–c**), the residential and commercial grid points are regarded as urban grid points. Black contour lines enclose areas of the new capital of Nusantara or IKN, which are divided into three sub-regions: KIPP for the central government (denoted by red line), KIKN for urban areas (between red and blue lines), and KPIKN for other development areas (between blue and black lines).

The new capital of Nusantara or IKN is being built on the eastern side of Kalimantan Island (Fig. 1). The WRF model provides a default land use/land cover (LULC) and vegetation fraction dataset that was processed from MODIS (Fig. 2a, d). However, the dataset is outdated and does not well represent the existing surface conditions. To update the land surface data, we modify the default LULC by incorporating present LULC information provided by the Nusantara National Capital Authority (OIKN) (Fig. 2b). OIKN also provides future LULC plan data of the new capital (Fig. 2c) for climate experiments with the fully built capital scenario. OIKN designed three sub-areas inside the capital: the government center area (KIPP), the urban center area (KIKN), and the development area (KPIKN). The presidential statutes No. 63 and No. 64 of 2022 provide detailed LULC plan for the capital^1,2^. Updated vegetation fractions are determined from lookup table of the WRF’s vegetation parameters (Fig. 2e, f).

Several climate datasets are used in this study. ERA5 reanalysis data^35^ is utilized for initial and boundary conditions of the regional climate model. It has 0.25° horizontal resolution with a 1 h interval. ERA5 includes 2-D surface climate parameters and 3-D multi-level atmospheric parameters. Previous studies have frequently used ERA5 as the input of regional climate modelling^36–38^. To ensure that the downscaling output agrees well with the observation in present climate, we evaluate the control simulation with surface observation operated by Indonesian Agency of Meteorology, Climatology, and Geophysics (BMKG). For conducting global warming projections with the PGW approach, we use five CMIP6 global model outputs (Table 1) to extract global warming signals that are required to modify initial and boundary conditions of the regional model. These CMIP6 models were often mentioned in previous papers as the best models in representing regional climate over Southeast Asia (Table 1). As for the projected socioeconomic global change scenario, we select SSP5-8.5 in order to simulate climate responses under the largest anthropogenic forcing^39^.

**Table 1:** List of CMIP6 models used for PGW simulation.

Model name	Institution/research center	Model resolution	Mentioned as best CMIP6 models in Southeast Asia by
E3SM-1–0	Lawrence livermore national laboratory, USA	1.000° × 1.000°	^40–42^
EC-Earth3	EC-earth consortium, Europe	0.703° × 0.703°	^41,43–47^
GFDL-ESM4	NOAA geophysical fluid dynamics laboratory, USA	1.250° × 1.000°	^44,46^
MPI-ESM1-2-HR	Max Planck institute for Meteorology, Germany	0.938° × 0.938	^42,48–50^
MRI-ESM2-0	Meteorological research institute, Japan	1.125° × 1.125°	^42,45^

### Simulation design

The simulations are carried out by using WRF model^51^, which has been widely used to provide regional weather/climate data for various applications (e.g.,^38,52–54^). The WRF model requires initial and boundary conditions of basic atmospheric parameters, typically obtained from global model output. Table 2 shows several key model configurations in our simulations. We utilize WRF version 4.2, which is set to have three-nested domains with horizontal resolutions of 9 km, 3 km, and 1 km, respectively (Fig. 1). Results from the innermost domain (Domain 3) are used in the analysis. The size of the outermost capital boundaries is approximately 80 × 50 km, with its governmental center (located in the western part) elongates about 40 km. With this size, the utilization of 1 km is arguably reasonable to reproduce mesoscale atmospheric conditions affected by the capital. Previous studies used similar resolutions to simulate urban atmospheric conditions in various cities around the world^55–59^.

**Table 2:** WRF model configuration used for simulation.

Configuration	Domain 1	Domain 2	Domain 3
Spatial resolution	9 km	3 km	1 km
Microphysics parameterization	WSM 3-class simple ice	WSM 3-class simple ice	WSM 3-class simple ice
Cumulus parameterization	Kain-Fritsch (new Eta)	Kain-Fritsch (new Eta)	–
Boundary layer parameterization	MRF	MRF	MRF
Shortwave radiation Parameterization	Dudhia	Dudhia	Dudhia
Longwave radiation Parameterization	RRTM	RRTM	RRTM
Urban canopy model (UCM)	–	–	Yes (single layer UCM)

Selected parameterization schemes used to estimate some atmospheric dynamics and physics are shown in Table 2. The first two domains utilize a cumulus parameterization scheme, but the third domain simulates the convection explicitly. We apply the single layer urban canopy model (SLUCM)^60,61^ for the third domain. SLUCM parameterizes the three-dimensional nature of urban surfaces by considering various surface physical properties within lowest parts of urban boundary layer, including wind profile and exchanges of radiation, heat, and momentum between buildings and land–atmosphere. SLUCM utilizes predefined urban canopy parameters for LULC categorized as low-intensity residential, high-intensity residential, and industrial/commercial (details of the parameters explained by^53^).

Table 3 summarize four simulations conducted in this study. HIST_noIKN describes present climate conditions in the absence of the new capital. LULC in Fig. 2b is used for HIST_noIKN. This simulation acts as a reference for quantifying differences in other simulations; hence it is also called control simulation (CTRL). Because UHI is more robust in sunny weather^6^, the simulation is run for one month when the rainfall climatology is minimum. Based on a nearby meteorological station data over 1991–2020, the lowest amount of rainfall and the minimum number of rainy days appear in September (Figure A.1 in Supplementary Information). To avoid the potential presence of climate variabilities, we check the occurrences of El Niño-Southern Oscillation (ENSO)^62^, Indian Ocean Dipole (IOD)^63^, and Madden–Julian Oscillation (MJO)^64,65^. ENSO and IOD are regarded as the two most prominent drivers of interannual climate variability in Indonesia (e.g.,^66,67^). Based on the well-known ENSO and IOD indicators, we find that ENSO and IOD phases are classified as neutral in September 2014^68,69^. MJO is a notable variability in intraseasonal scale^70,71^, which was mostly inactive in that month^72^. Therefore, to conduct HIST_noIKN, we choose September 2014 as simulation period. The model uses time-integration from 00UTC 31 August to 00UTC 1 October 2014 and the first day is discarded due to spin-up time. The initial and boundary conditions are obtained from ERA5 reanalysis.

**Table 3:** List of four model experiments carried out in this study.

Name	Description	LULC data	Initial and boundary condition
HIST_noIKN (CTRL)	To describe current state of climate, regarded as the control simulation	Present	ERA5
HIST_IKN	With LULC modification. To quantify the effect of urbanization under present climate	Present + IKN modification	ERA5
FUTR_noIKN	Future projection without LULC modification. To quantify the effect of global warming without urbanization	Present	PGW = ERA5 + $\Delta$
FUTR_IKN	Future projection with LULC modification. To quantify the effect of both global warming and urbanization	Present + IKN modification	PGW = ERA5 + $\Delta$

HIST_IKN experiment quantifies atmospheric responses to urbanization when the background climate is unchanged (i.e., urban effect without global warming). It uses a modified LULC scenario where the new capital is assumed to be fully established following the government’s plan (Fig. 2c). IKN is designed to have 67.75% land for protected areas, including forests and city parks. Combined with crops areas, IKN will have a total of 84.14% green fraction over the whole capital. For a comparison, other cities in Indonesia such as Jakarta and Denpasar were reported to have green fractions of 4.65% and 36.28%, respectively^73^. Large green fractions theoretically will suppress the effects of temperature increase and thus urban heat island^6^. However, the green fraction of Nusantara is mostly attributed to the isolated forest areas in the central part (KPIKN) (Fig. 2c, f). Urban temperature rise is hypothesized to still emerge in the western part where the center of capital resides (KIPP and KIKN).

FUTR_noIKN and FUTR_IKN experiments consider the effects of global warming. The simulations are conducted by performing the PGW approach^33,34^. In recent years, PGW simulations have been frequently used in research as an alternative regional climate modeling strategy to the conventional long-term downscaling^17,74–76^. In a PGW simulation, future changes in the climate system are directly imposed by modifying the regional model’s initial and boundary conditions, which are assumed to be a linear coupling of the reanalysis/observation and the differences of the global warming estimated by GCMs. Therefore, FUTR experiments are basically similar to HIST experiments except that their initial and boundary conditions are constructed by adding ERA5 to climate change delta (∆):

$${\text{PGW}} = {\text{ERA}}5 + \Delta$$

∆ is computed by calculating differences of GCM parameters averaged over future period (${\text{GCM}}_{futr}$) and historical period (${\text{GCM}}_{hist}$):

$$\Delta_{m} = {\text{GCM}}_{futr,m} - {\text{GCM}}_{hist,m}$$

The averaging is done separately each month $m$ to distinguish changes in different seasons. In this study, we define the future and historical periods as 2051–2060 and 2005–2014, respectively. We select the future period of mid-twenty-first century because the government aims to accomplish the capital in 2045^1,2^. ${\text{GCM}}_{futr}$ and ${\text{GCM}}_{hist}$ are constructed by calculating ensemble means of the selected five GCMs shown in Table 1. $\Delta$ is calculated for the following climate parameters: three-dimensional geopotential height, air temperature, horizontal wind velocity, humidity; and two-dimensional surface skin temperature and soil temperature.

## Results

### Model evaluation in present climate

Temperature estimations of ERA5 and WRF output in September 2014 are evaluated by using hourly temperature observation in a nearby meteorological station (Table B.1 in Appendix B). The correlation coefficient of WRF is 0.77, slightly better than ERA5 (0.70). Mean absolute errors (MAE) of WRF and ERA5 are respectively 1.66 °C and 1.26 °C, largely attributed to cool biases as shown by negative mean errors (ME). Despite WRF showing larger MAE than ERA5, WRF simulates much better diurnal range of temperatures. MAE of WRF in depicting diurnal range is 0.67 °C, while for ERA5 is 2.83 °C. We also evaluate the model in monthly mean viewpoint as our analyses are mostly based on monthly composites. The correlation coefficient of WRF temperature for monthly mean diurnal cycle is 0.92, greater than ERA5 (0.87). For diurnal range difference of monthly mean diurnal cycle between model and observation, WRF slightly overestimates the diurnal range (0.19 °C) but ERA5 shows large underestimation (− 2.65 °C).

The evaluation result confirms that the regional model of WRF generally performs better than the global reanalysis of ERA5. It should be noted that the referenced meteorological site is just next to an international airport (Figure B.1 in Supplementary Information). This heat source is located in the upwind side. It is one possible reason for cool biases in the model as the 1-km resolution may not have adequately simulated smaller temperature patterns. Multiple and dense observation points over a wider area are more desirable for future evaluation.

### Climate projection of temperature and urban heat island (UHI)

**Fig. 3:**
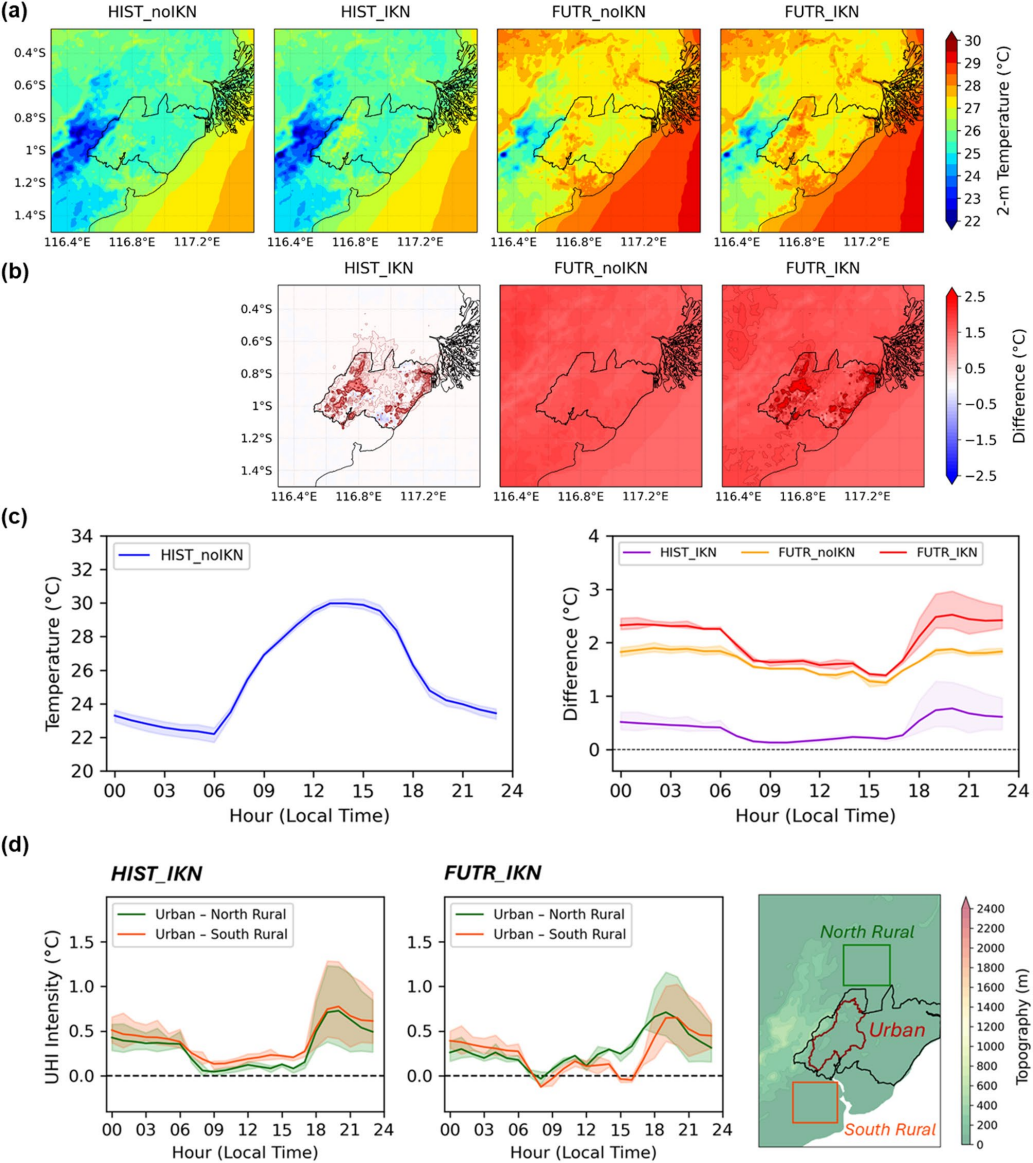
Simulated 2-m air temperature and UHI. (**a**) Daily means of spatial temperatures from the four simulations. (**b**) differences of the three experiments with the control experiment (HIST_noIKN). From left to right the panels represent the urban effect (HIST_IKN), the global effect (FUTR_noIKN), and the urban + global effects (FUTR_IKN), respectively. (**c**) Left panel shows mean diurnal temperature variation of central urban areas of HIST_noIKN (denoted as red contour in the right panel of (**d**)). Right panel of (**c**) shows mean temperature increases of the urban areas in the three experiments. (**d**) Diurnal variation of UHI intensity calculated by urban–rural differences in HIST_IKN (left panel) and FUTR_IKN (middle panel) experiments. The locations of north rural and south rural are shown in the right panel. Shadings in the time series of (**c**–**d**) indicate variations among grid points in the designated polygons, denoted by 25th and 75th percentiles.

Figure 3a shows spatial variations of 2-m air temperature simulated by the model. In the western side of the domain the temperature is relatively cooler because of high topography (Fig. 1). To the south of IKN around 1.2°S on the coastal side, there is a clear warmer area, showing an existing urban heat island due to the city of Balikpapan. In the control run (HIST_noIKN), there are no obvious warm areas found in the new capital region (Fig. 3a first panel). Temperature increases in the new capital are gradually seen in the added urban and global warming simulations (HIST_IKN, FUTR_noIKN, and FUTR_IKN) (second to fourth panels in Fig. 3a).

Temperature differences between the experiments and the control run quantify the impacts of urban effects (HIST_IKN), global effects (FUTR_noIKN), and urban + global effects (FUTR_IKN). The differences are shown in Fig. 3b. As the capital is assumed to be fully built in the present climate (HIST_IKN), the model estimates a clear temperature increase in the western side of IKN to the responses of urban surfaces in KIPP and KIKN, areas where the government and city activities mostly reside. The isolated temperature increases denote the new capital UHI, which appears to extend slightly northward. In the eastern side of IKN, some temperature increases also appear near the coastline because this area also has land modification plans, which are mostly related to agriculture (Fig. 2c). Under global warming influence without IKN, the increase happens but it is very homogenous across the simulation domain regardless of the surface conditions (FUTR_noIKN). When the capital is finished in the future, the combined impacts of land surface modification and global warming yield a much warmer temperature inside of the IKN (FUTR_IKN). The spatial pattern of temperature increase in FUTR_IKN looks similar with that in HIST_IKN, indicating linear responses to LULC change and global warming.

In the diurnal viewpoint, Fig. 3c (right panel) shows that the monthly mean temperature increases in urban areas of IKN are larger in nighttime (18–06 LT) than in daytime (06–18 LT). This is consistent with the common knowledge of urban effects^6^. The large positive difference in the nighttime temperature is typically because urban areas fail to cool as rapidly as rural areas owing to trapped outgoing radiation and anthropogenic heat emission. By averaging grid points in KIPP and KIKN, the increase in urban air temperature ranges from 0.13 to 0.78 °C for HIST_IKN, 1.24 to 1.90 °C for global warming with SSP-5.85 scenario, and 1.37 to 2.54 °C for FUTR_IKN simulation. In general, urbanization exerts a less pronounced impact compared to global warming. The degree of warming inside KIPP and KIKN varies due to mixed patches of urban and non-urban grid points (Fig. 2c). To reveal the highest urban effects, we find a daily maximum temperature increase from grid points in these regions. The maximum increases reach 2.89 °C and 4.47 °C for HIST_IKN and FUTR_IKN simulations, respectively.

We calculate the intensity of UHI by subtracting the area averaged urban temperature with temperatures in rural areas at similar topographic levels (Fig. 3d). The UHI intensity is also largest at nighttime, especially in the evening. During daytime, UHI intensity is relatively weak and unclear. The lack of UHI at this period was also found in previous studies as summarized in^6^. These can be affected by slow warming in early daytime due to shade in urban canyons and high thermal admittance of urban surfaces. In respect to north rural area, the UHI intensity is generally smaller than UHI calculated with the south rural area (Fig. 3d, left panel).

To compare the UHI of IKN with that of the nearest existing city, we calculate the UHI intensity of Balikpapan, using the rural area to the west of the city as the reference and based on the output of the control run (HIST_noIKN) (Figure A.2 in the Supplementary Information). We find that the average UHI in Balikpapan is approximately 1.5 °C and can exceed 2 °C. This is greater than the UHI in IKN (Fig. 3d). Since Balikpapan is an older city that was developed with relatively poor spatial planning, this result highlights the benefits of the “green design” planned for the new capital.

### Changes in UHI under different wind

**Fig. 4:**
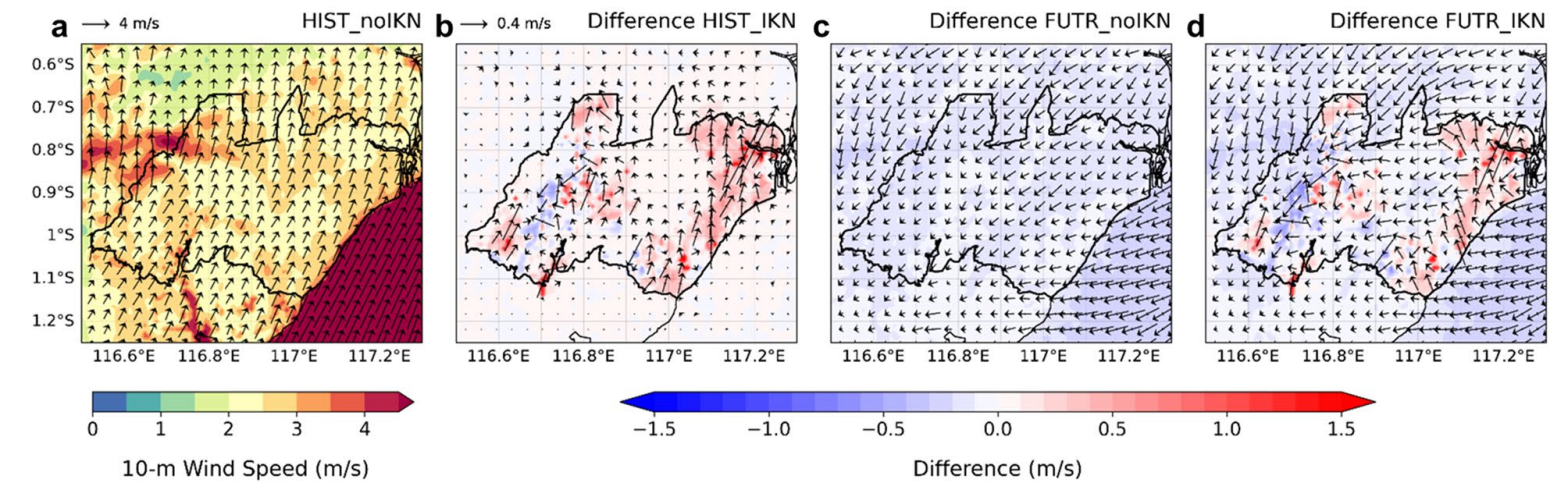
(**a**) Mean 10-m wind speed (shading) and vector field (arrows) simulated in HIST_noIKN (left panel). (**b**-**d**) show differences in wind speed and wind vector compared to (**a**) for HIST_IKN, FUTR_noIKN, and FUTR_IKN, respectively.

The magnitude and extent of UHI depend on several meteorological factors, especially wind. As shown in Fig. 3b, the rural areas north of IKN appear to be affected by increasing temperature from the capital, indicating urban heat advection by wind. This is consistent with the background wind conditions, which show a prevailing southerly pattern in Fig. 4a. As a result, the city-rural temperature difference is weaker on the northern side compared to the southern side of IKN (Fig. 3d, left panel). The southerlies during this month are seasonally driven by Asian summer monsoon and Australian winter monsoon systems^77,78^.

In the presence of IKN (HIST_IKN), the model simulated a mixed pattern of increases and decreases in wind speed, but the changes are much lower than the original wind speed, indicating that the southerlies persist despite IKN’s development (Fig. 4b). Similar noisy signals in wind speed changes due to urban effects were also documented in a urban climate study of the Jakarta^17^. Although decreases in wind speed are widely expected because of increased surface roughness, the reduction is only observed in the western part of the KIPP/KIKN. Along the coastal side of IKN, the conversion of forest areas into croplands leads to strengthening of wind speed due to changes in aerodynamic parameters.

Given the evident influence of wind on UHI under the present climate, it is important to examine potential changes in wind patterns under future global warming. The projected background southerlies consistently weaken in the future climate (Fig. 4c), and the presence of IKN further enhances this weakening over the capital area (Fig. 4d). The reduction in wind speed reaches up to 0.5 m/s, which could significantly suppress temperature advection to the north. This condition likely explains why the magnitude of UHI to the north does not differ significantly from that to the south under future climate projections (Fig. 3d, second column).

**Fig. 5:**
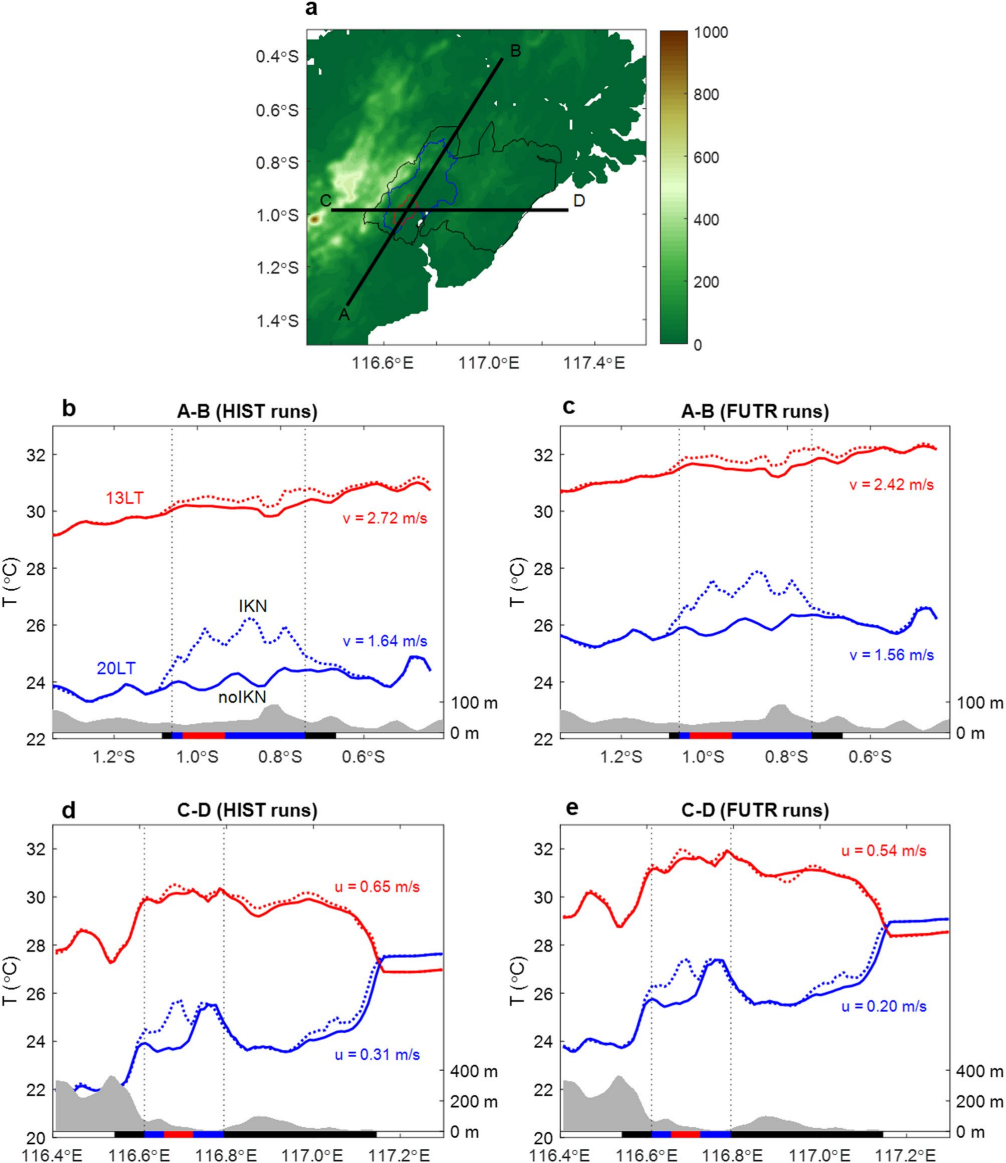
Line transects of surface air temperatures along A-B and C-D lines depicted in (**a**). Shading indicates topography. (**b**) and (**c**) shows temperature patterns along A-B line for HIST and FUTR simulations, respectively. Solid and dashed lines are based on simulations without and with IKN, respectively. Red and blue lines denote temperatures at 13LT and 20LT, respectively. Texts in the right side of (**b**-**c**) show mean meridional wind speed along the transect. Grey shading in the bottom denotes topography with its scale shown on the right axis. Horizontal thick black, blue, and red lines indicate development areas of IKN (KPIKN), urban areas of IKN (KIKN), and government center of IKN (KIPP), respectively. (**d**) and (**e**) are same with (**b**) and (**c**), except for transect along C-D line. Texts in the right side of (**d**–**e**) show mean zonal wind speed along the transect.

Wind speed varies throughout hours and days depending on weather conditions. To further investigate the impact of wind speed variations on UHI, we perform transect analyses on two designated lines as shown in Fig. 5a. We compare UHI structures along southwest-northeast (A–B) and west–east (C–D) at daytime (13 LT) and nighttime (20 LT) for historical and future simulations. The A-B transect line aligns well with the axis of mountains in the west, and the direction of background winds, which are dominated by southerlies (Fig. 4a). During the daytime, the presence of urban areas yields slight warming above KIKN, which extends far north in the downwind areas (Fig. 5b, red dashed line). A robust warming resembling an “island” appear in the nighttime (Fig. 5b, blue dashed line). The excess of heat seems to also affect the downstream area but with a more limited region, possibly owing to the weaker southerlies during the nighttime compared to the daytime. Under global warming effects (Fig. 5c), the UHI feature looks similar, but its magnitude is somewhat suppressed although the background temperature increase is quite clear.

The west–east transect of temperatures shows a more complicated feature of UHI (Fig. 5d,e) due to diverse LULC changes, topography, and land-sea distribution. Zonal wind values along the transect indicate westerly winds but they are very weak, limiting the extension of temperature increases. In the nighttime, the western UHI is attributed to urban areas of KIPP and KIKN but its surrounding temperature is largely affected by high mountains in the west of IKN and wide basin in the east of IKN, resulting in cooler and warmer background temperatures, respectively. In the coastal side, the eastern UHI appears due to the conversion from forest areas into few urban patches and large croplands (Fig. 2c). However, its UHI magnitude is difficult to quantify because of the effect of warmer nearby seas. In future with global warming (Fig. 5e), the spatial patterns are generally consistent, especially in the nighttime.

**Fig. 6:**
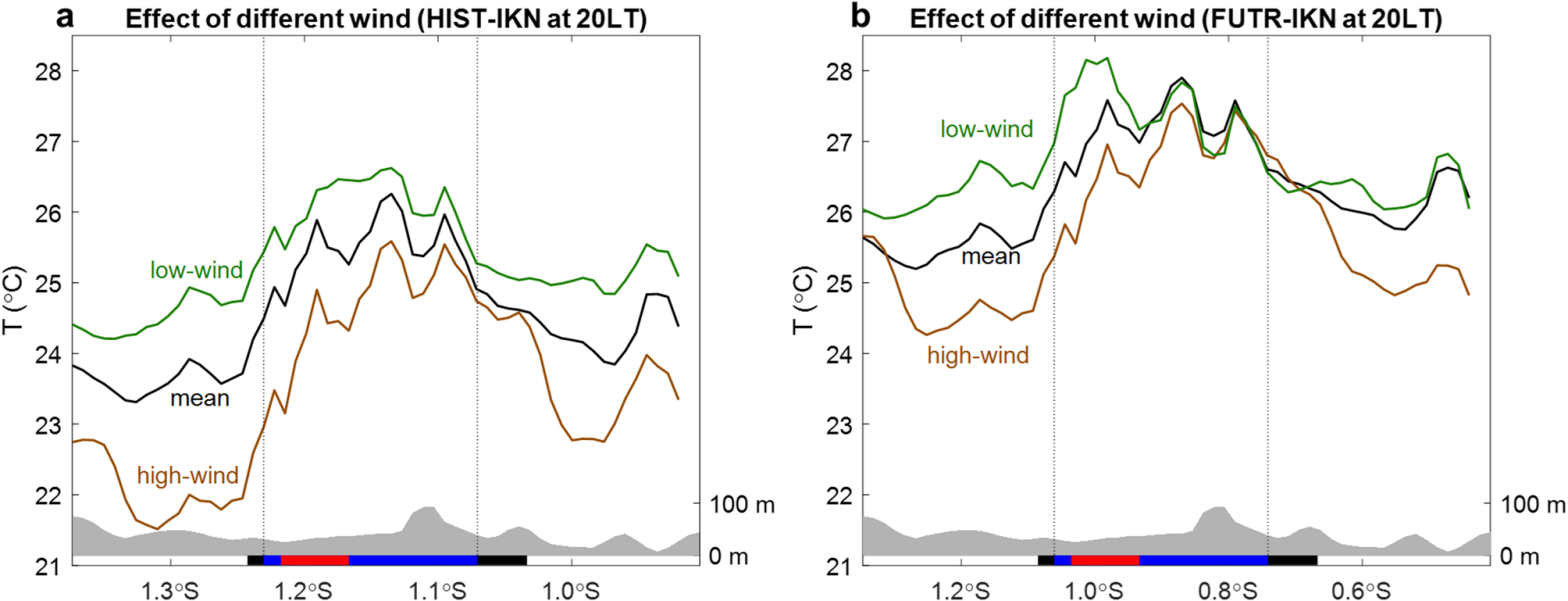
(**a**) and (**b**) are similar to panels (**b**) and (**c**) in Fig. 5, except for different background wind speeds. Green and brown lines denote simulated temperature under low wind and high wind conditions, respectively. Only temperature features at 20LT are shown.

To understand the impact of different magnitude of wind speed on the temperature and UHI, we compare temperature patterns along the A-B transect during high wind and low wind conditions (Fig. 6), which are determined based on the highest and the lowest daily-mean wind speeds from the 30-day simulation, respectively. The high wind condition is expected to decrease the temperature (Fig. 6a) due to enhanced vertical mixing, evaporative cooling, and urban heat ventilation^6^. The temperature decrease is significantly detected from the upwind south of IKN up to northern IKN. In contrast, the low wind weather causes temperature increases along the transect line. Previous studies noted that the impact of winds on urban heat is more profound in low-latitude areas and under hot weather conditions^79–81^. Under future global warming effect, the FUTR-IKN simulation shows that the increases and decreases in temperatures are not as large as those in the HIST-IKN.

## Discussion

The current study provides useful information for spatial planners and regulators to evaluate the current grand design of the new capital for a sustainable and livable city in future. To potentially reduce UHI impact in the city, Nature-based solutions or ecosystem-based approaches were identified to play a crucial role and will become even more important under the changing climate^82,83^. As Nusantara Capital Authority also plans to implement Nature-based Solutions by enhancing green and blue spaces^84^, understanding the potential UHI characteristics and detecting the areas may become beneficial to proper spatial planning for blue and green spaces in the future. A recent study has numerically shown the benefit of enlarging the blue-green spaces on urban heat reduction in KIKN^85^. As the current study shows that UHI created by IKN can affect neighboring villages and cities, the spatial planning for green and blue spaces of the surrounding cities, such as Samarinda, Penajam Paser Utara, or Balikpapan, are encouraged to be considered and well-aligned with the Nusantara spatial plan. Regulations for built-up settings and spatial planning are not only necessary for the megacity but also its satellite towns^86^. This issue will likely be relevant to Nusantara and its surrounding cities which potentially become its satellite cities. Hence, it is important to also prioritize the enhancement of the blue and green space for those cities. For a more local view inside the city, further studies may look at more detailed urban planning to assess cooling potential such as precinct ventilation^87^.

The above findings are produced numerically in the model and must contain some errors. To capture the real condition of temperature changes in future, many meteorological observation points are necessary to be deployed in and around IKN. The observed data is also critical to model evaluation and improvement in future. This study is conducted from one-month simulations because of limitation in computational resources. We selected the month of September because its monthly rainfall is minimum throughout the climatological year, and thus providing favorable conditions for UHI. Although tropical regions do not exhibit distinct seasons throughout the year (i.e., summer–winter cycle), there may still be variations of UHI between seasons. For example, during the Asian winter monsoon in December to February, the background wind is mostly northerlies^88^. Therefore, the spatial extension of urban temperature may switch to southward. In transitional seasons such as October and April, the wind speed is relatively lower, and this condition may yield to a more robust UHI if sunny days persist. Future studies can confirm these speculations and provide more comprehensive documentation on UHI in IKN.

As urban temperatures and UHI are affected by background weather conditions, it is interesting to study how UHI behaves under different well-known synoptic forcing such as equatorial waves, Madden–Julian Oscillation, El Nino-Southern Oscillation, and Indian Ocean Dipole. The superposition of compounded phenomena may lead to some extreme events, which are critical for heat stroke risk assessment. A recent study has evaluated performance of a mesoscale model to reproduce extreme urban heat in Jakarta^89^. A similar approach can be done to IKN area.

## Conclusion

This study investigates near-surface air temperature changes in response to urbanization and global warming in Indonesia’s new capital, Nusantara (IKN). Using updated official LULC data incorporated into a mesoscale climate model with a pseudo-global-warming approach, we assess the relative contributions of urbanization and future global climate change to temperature increases over the region.

The results show that global warming exerts the dominant influence, with spatially averaged temperature increases of 1.24–1.90 °C across IKN. By comparison, urbanization-induced warming is weaker, with increases ranging from 0.13 to 0.78 °C, particularly at night. When both global warming and urbanization are considered, the average temperature in urban areas is projected to rise by 1.37–2.54 °C, with the hottest grid point reaching an increase of 4.47 °C. The strongest warming occurs in the western part of IKN, where government offices and urban centers are planned. In contrast, the central part of IKN shows relatively little warming due to preserved forest areas, while the eastern coastal zone experiences moderate warming associated with land conversion from forests to urban patches and croplands.

The spatial pattern of warming resembles an isolated UHI. The UHI extends northward under southerly wind advection, affecting rural areas north of IKN. The meridional UHI pattern is more robust than the zonal one, and UHI intensity is particularly strong at night, averaging 0.7 °C between the western urban core and surrounding rural areas. Changes in background wind speed further modulate both the magnitude and spatial coverage of urban warming, emphasizing the varying risks of extreme heat under different wind conditions.c

This study confirms that the urbanization effect is less pronounced that the global warming effect, in contrast to a similar study conducted in Jakarta^17^. The weaker urban warming may reflect the benefits of IKN’s planned green design, which incorporates significant green space and vegetation patches. Nevertheless, both factors contribute to higher temperatures in future IKN. Further impact studies are needed to assess potential heat-related hazards and risks in the new capital.

## Supplementary Information

Below is the link to the electronic supplementary material.

Supplementary Material 1

## Data Availability

Data will be made available on request addressed to the corresponding author.
