# Supplementary material for: Urbanization and global warming impacts of Indonesia’s future capital of Nusantara on air temperature and urban heat island
Source: Sci Rep. 2025 Nov 24;15:41543. doi: 10.1038/s41598-025-25500-8 (PMC12644725; doi:10.1038/s41598-025-25500-8)
Supplement: Supplementary file 1 — Supplementary Material 1 [file 41598_2025_25500_MOESM1_ESM.docx]

# Supplementary Information for “Urbanization and global warming impacts of Indonesia’s future capital of Nusantara on air temperature and urban heat island”

## A. Monthly climatology of observed rainfall


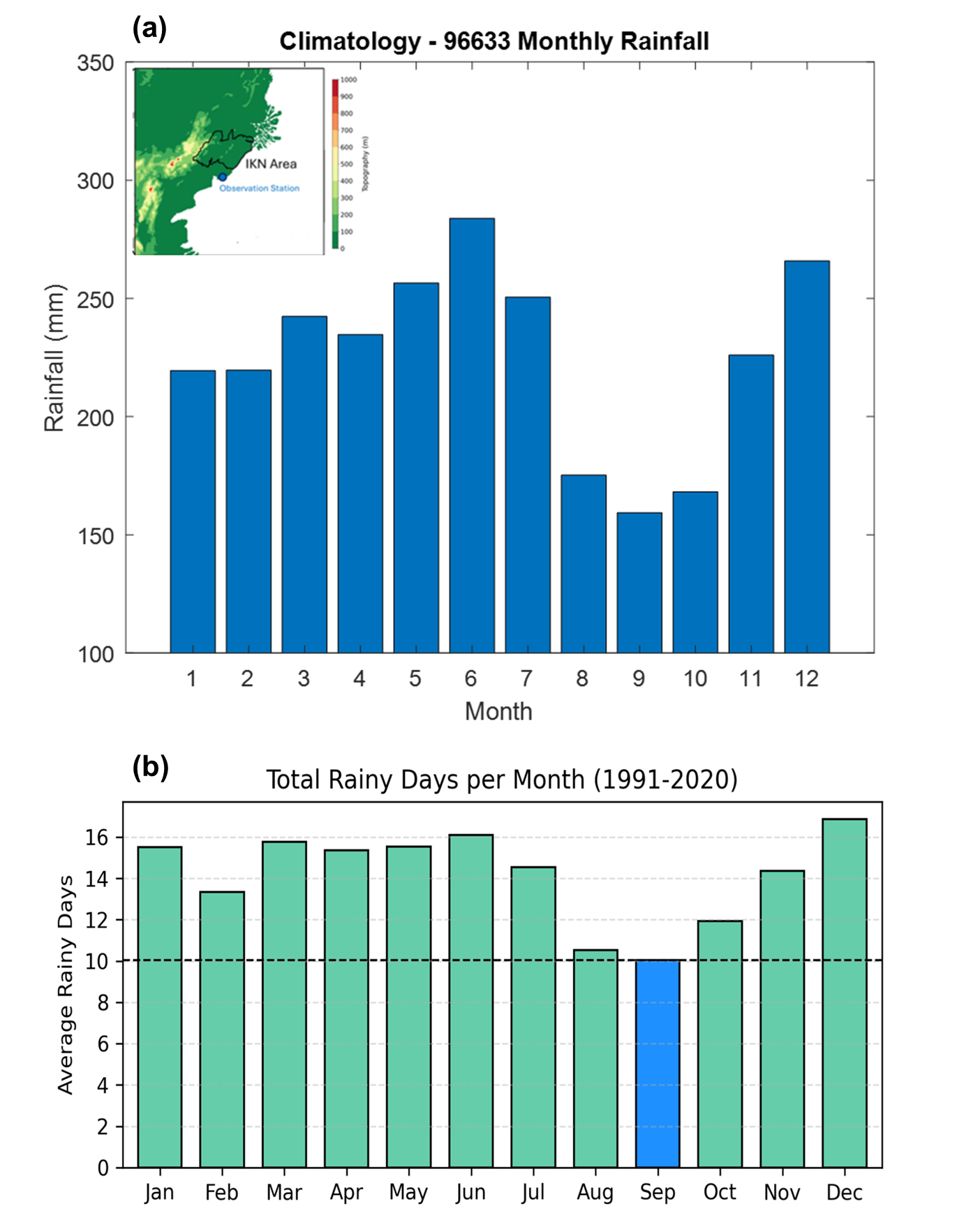


**Figure A.1.** Annual cycle of monthly (a) total rainfall amount and (b) number of rainy days in a meteorological station close to the new capital (location shown in the top left). The climatological period is 1991-2020.


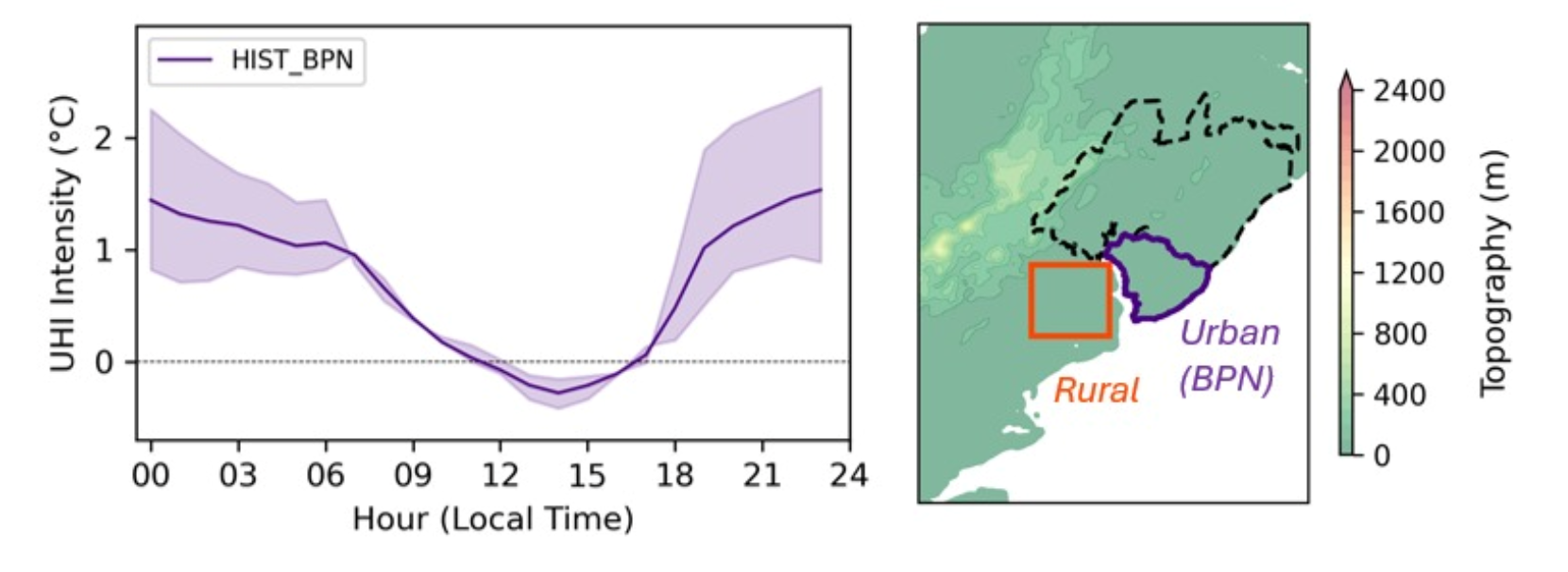


**Figure A.2.** Monthly average of hourly UHI intensity calculated based on Balikpapan city (BPN; purple contour in right panel) and rural area in the west of BPN. This is calculated from control run (HIST_noIKN).

## B. Model evaluation

**Table B.1**. Evaluation of reanalysis temperature (ERA5) and downscaled temperature (WRF domain 3) in September 2014. The parameter is hourly near-surface temperature at 2 m. The location of meteorological station is shown in Fig. A.1 and Fig. B.1.

| Indicator | ERA5 | WRF (domain 3) |
| --- | --- | --- |
| Correlation coefficient (*r*) | 0.70 | 0.77 |
| Mean absolute error (MAE) and mean error (ME) | 1.26°C and -0.87°C | 1.66°C and -1.50°C |
| MAE and ME of diurnal range ($\boldsymbol{\equiv}\boldsymbol{T}_{\boldsymbol{max}}\mathbf{-}\boldsymbol{T}_{\boldsymbol{min}}$) | 2.83°C and -2.83°C | 0.67°C and -0.23°C |
| *r* of monthly mean diurnal cycle | 0.87 | 0.92 |
| Error of diurnal range in monthly mean diurnal cycle | -2.65°C | 0.19°C |


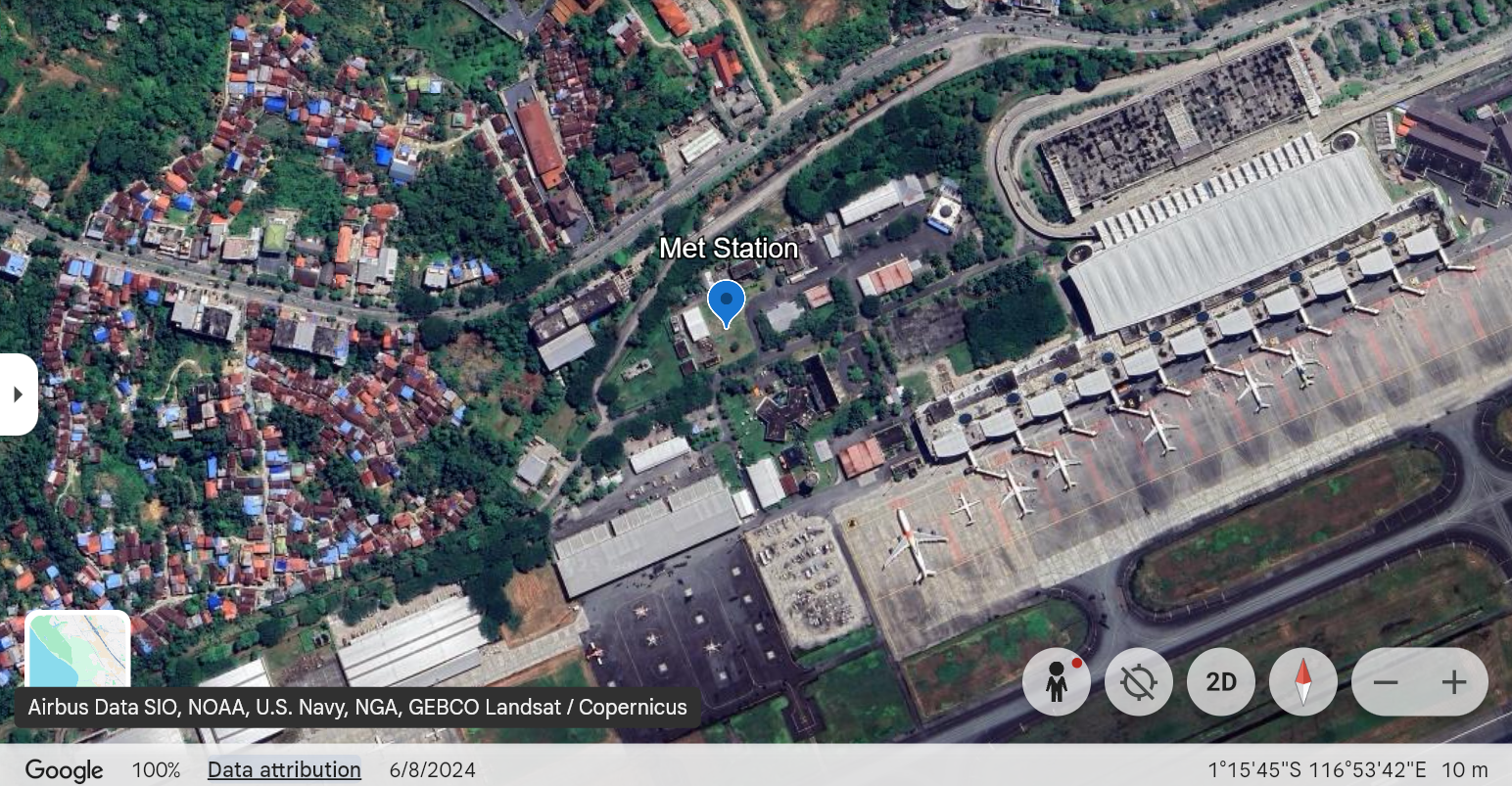


**Figure B.1.** Detailed location of meteorological station, which is located in the north of international airport in Balikpapan city. The image is obtained from Google Earth with data sources from Airbus Data SIO, NOAA, U.S. Navy, NGA, and GEBCO Landsat/Copernicus.
